# Comparison of Fibronectin and Collagen in Supporting the Isolation and Expansion of Endothelial Progenitor Cells from Human Adult Peripheral Blood

**DOI:** 10.1371/journal.pone.0066734

**Published:** 2013-06-18

**Authors:** Elena Colombo, Francesca Calcaterra, Monica Cappelletti, Domenico Mavilio, Silvia Della Bella

**Affiliations:** 1 Lab of Clinical and Experimental Immunology, Humanitas Clinical and Research Center, Rozzano (MI), Italy; 2 Department of Medical Biotechnologies and Translational Medicine, University of Milan, Milan, Italy; University of Padova, Italy

## Abstract

**Background:**

Endothelial colony-forming cells (ECFCs), are circulating endothelial progenitor cells increasingly studied in various diseases because of their potential for clinical translation. Experimental procedures for their ex vivo culture still lack standardization. In particular two different extracellular matrix proteins, either fibronectin or collagen, are commonly used by different Authors for coating plastic plates, both allowing to obtain cells that have all the features of ECFCs. However, possible differences in the impact of each substrate on ECFCs have not been analysed, so far. Therefore, in this study we investigated whether fibronectin and collagen may differentially affect ECFC cultures.

**Methodology/Principal Findings:**

ECFCs were isolated and cultured from peripheral blood mononuclear cells of healthy donors. The impact of fibronectin compared with collagen as the only variable of the experimental procedure was analysed separately in the phase of isolation of ECFC colonies and in the following phase of cell expansion. In the isolation phase, although similar frequencies of colonies were obtained on the two substrates, ECFC colonies appeared some days earlier when mononuclear cells were seeded on fibronectin rather than collagen. In the expansion phase, ECFCs cultured on collagen showed a longer lifespan and higher cell yields compared with ECFCs cultured on fibronectin, possibly related to the higher levels of IL-6 and IL-8 measured in their supernatants. ECFCs cultured on both substrates showed similar immunophenotype and ability for in vitro tube formation.

**Conclusions/Significance:**

Overall, the results of this study indicate that, although both fibronectin and collagen efficiently sustain ECFC cultures, each of them brings some advantages within individual steps of the entire process. We suggest that colony isolation performed on fibronectin followed by cell expansion performed on collagen may represent a novel and the most efficient strategy to obtain ECFCs from adult peripheral blood samples.

## Introduction

Endothelial progenitor cells (EPCs) are bone marrow-derived cells playing a critical role in adult vasculogenesis and endothelial homeostasis [1], [2]. Similar to embryonal angioblasts, EPCs are recruited from the bone marrow to sites of endothelial injury, where they contribute to blood vessel formation and repair through their ability to proliferate, differentiate into mature endothelial cells, integrate into the endothelial wall and produce proangiogenic cytokines [3]–[5]. EPCs have stimulated considerable interest in recent years due to the observation that variations in their number and function are associated with many pathological conditions, mainly cardiovascular diseases and cancer [6]–[12]. The behaviour of EPCs during particular physiologic challenges are under intense investigation as well, to better understand the role and function of these cells in human physiology [9], [13], [14]. Moreover, EPCs are being studied for their role in maintaining the integrity of endogenous endothelium and their regenerative potential, with studies indicating the beneficial effect of EPC-based cell therapies in both animal models and human diseases [15], [16].

Circulating EPCs can be studied following two main approaches [17]. One approach consists in identifying and selecting EPCs by cell surface phenotype using fluorescently labeled antibodies and flow cytometry directly performed on peripheral blood samples. Key advantages of this strategy reside in need for very small blood samples, rapidity of execution and providing results that more directly reflect the in vivo situation, compared with other methods that investigate EPCs from blood samples after in vitro adhesion and growth. The second approach consists in isolating and expanding EPCs in vitro, starting from peripheral blood samples. Compared with the first approach, this strategy offers the advantage to allow more detailed cell characterization and obtain cells in sufficient numbers to be used in animal experimental models and in possible EPC-based cell therapies.

Among populations of putative EPCs that can be isolated and cultured from adult blood, only endothelial colony-forming cells (ECFCs), also called late-EPCs or outgrowth endothelial cells, are considered the true EPCs. In fact, they have all the characteristics of true endothelial progenitors, as they possess clonal proliferative potential and they express endothelial but not hematopoietic cell surface antigens. Most important, unlike other putative EPCs that are endowed with proangiogenic activity but fail to display vasculogenic activity, ECFCs have the unique ability to display in vivo vasculogenic activity when implanted into immunodeficient mice [18], [19]. Because of the potential for clinical translation, ECFCs isolated and propagated from peripheral blood are a promising cell source for many applications including extensive characterization in an increasing number of diseases, as well as in vivo vasculogenesis and regenerative medicine.

Procedures for ECFC isolation and propagation strictly depend on coating the culture surface with extracellular matrix protein that promotes cell adhesion and growth [20]. In particular, either fibronectin [21]–[26] or collagen [27]–[32] are commonly used as cell culture substrates by different research groups to successfully isolate and propagate cells that fulfill the criteria to be defined ECFCs. However, possible differences in the impact of each substrate on ECFC cultures have not been analysed, so far. Therefore, in this study we investigated whether fibronectin and collagen may differentially affect ECFC culture. To this aim, we directly compared the effects of fibronectin and collagen, analysing separately the two phases of the entire process: the phase of isolation of ECFC colonies and the subsequent phase of ECFC cell expansion. In this way, we could demonstrate advantages of each substrate within individual steps of ECFC culture, and suggest that the sequential use of both substrates may represent a novel and the most efficient strategy to obtain ECFCs from adult peripheral blood samples.

## Materials and Methods

### Ethics statement

Ethics approval was obtained from the Institutional Review Board of Istituto Clinico Humanitas, Rozzano, Milan, Italy, and written informed consent was provided by study participants.

### Isolation and culture of ECFCs

ECFCs were isolated and cultured from peripheral blood mononuclear cells (PBMCs) of healthy donors according to methods previously described [33], with some modifications. Briefly, PBMCs obtained by Ficoll density gradient centrifugation (Cedarlane, Hornby, Canada) were resuspended in EGM-2 medium (Cambrex Bio Science, Walkersville, MD) and seeded onto 24-well tissue culture plates (5×10^6^ cells/well) precoated with either human fibronectin (1 µg/cm^2^, Sigma-Aldrich, St. Louis, MO) or type I rat-tail collagen (5 µg/cm^2^, BD Biosciences, San Jose, CA), as indicated. After one day of culture, nonadherent cells and debris were aspirated, adherent cells were washed with EGM-2 medium, and EGM-2 medium was added to each well. Medium was changed every 2 days until the first passage. The initial appearance of visible colonies, identified as well circumscribed monolayers of cobblestone-like cells, was determined by daily culture observation using an inverted microscope, and the first day of ECFC colony emergence was recorded. Colony size was determined by measuring colony diameters 3 days after colony appearance. The frequency of ECFC colonies was determined by measuring the number of colonies in the primary culture on day 30, as no ECFC colony emerged at a later time point. ECFC colonies were released from the original tissue culture plates by trypsinization (trypsin 0.25%) (Euroclone, Wetherby, UK), resuspended in EGM-2 medium, and plated onto 6-well tissue culture plates precoated with fibronectin or collagen, as indicated. Subconfluent cells were further subpassaged in flasks and expanded until cell senescence, as determined by morphology changes, decrease in proliferation and positive staining for senescence-associated ß-galactosidase (BioVision Reasearch Products, Mountain View, CA) [34]. At each passage, viable cell counts were obtained using a hemacytometer and trypan blue exclusion, and cell-free culture supernatants were filtered and stored at –20°C.

### ECFC proliferation assay

Cell proliferation of ECFCs was assessed as described elsewhere [35], with some modifications. Briefly, after 16 h of serum and growth factor privation, ECFCs were incubated for 48 h in EGM-2 medium, supplemented with rhIL-6 and/or rhIL-8 (Peprotech, London, UK), as indicated. In inhibition experiments, neutralizing anti-IL-6 and anti-IL-8 mAbs (R&D Systems, Minneapolis, MN) were added 30 minutes before starting 48 h incubation with complete medium and serum. The concentrations of mAbs were chosen according to the Manufacturer’s instructions. Proliferation was examined after 48 h by the colorimetric Crystal Violet (CV) assay, as described elsewhere [36] with some modifications. Briefly, the cells were fixed with 4% paraformaldehyde and incubated in 0.1% CV for 20 min at room temperature. Excess dye was removed by three brief rinses with double-distilled water, the plates were air dried, and the dye was extracted with 10% acetic acid, which was then read in a plate reader at 550 nm.

### Cytokine measurement in ECFC supernatants

The amount of vascular endothelial growth factor (VEGF), basic fibroblast growth factor (bFGF), IL-6 and IL-8 in culture supernatants were determined by DuoSet ELISA kits from R&D Systems. All individual steps were performed according to the manufacturer’s instructions.

### Immunophenotyping of ECFCs

Immunophenotyping of ECFCs was performed as previously described [33]. For antigen detection by flow cytometry, FITC-, PE-, or PerCP-Cy5.5-conjugated monoclonal antibodies directed against anti-human CD14, CD31 (Becton-Dickinson); CD45 (e-Bioscience); VEGF receptor-2 (VEGFR-2) (R&D Systems); CD54, CD144 (Serotec); CD146 (Biocytex) were used. Isotype-matched irrelevant mAbs were used as negative controls. Data were acquired on a FACSCanto flow cytometer (Becton-Dickinson) and analysed using FlowJo 9.6.2 software (Tristar, Ashland, OR). Cells were electronically gated according to light scatter properties to exclude cell debris.

### Uptake of Dil-acetylated low-density lipoprotein and staining for Ulex Europeus lectin

The uptake of Dil-acetylated low-density lipoprotein (Dil-ac-LDL) by ECFCs and their staining for Ulex Europaeus Agglutinin-1 (UEA-1) were assessed as previously described [33]. Briefly, ECFCs were cultured onto fibronectin- or collagen-coated, 2-chamber Lab-Tek slides (Nalge NUNC International, Rochester, NY) and attached cells were incubated with 10 µg/ml Dil-ac-LDL (Molecular Probes, Eugene, OR) in EGM-2 medium for 1 hour at 37°C. Cells were then fixed with 2% formaldehyde for 10 minutes and incubated for 1 hour with FITC-labeled UEA-1 (Sigma-Aldrich). Slides were mounted in anti-fading mounting media containing DAPI (Vectashield; Vector Laboratories, Burlingame, CA) and analysed with an Olympus Fluoview FV1000 laser scanning confocal microscope.

### Matrigel capillary tube formation assay

Matrigel assays were performed as previously described [33]. Briefly, ECFCs were seeded onto 96-well tissue culture plates coated with 70 µl Matrigel (Becton-Dickinson) at a cell density of 2×10^4^ cells per well, and incubated at 37°C. Cells were observed during the following 12–72 hours with inverted microscopy for capillary-like formation.

### Statistical analysis

Data were shown as mean ± standard error of the mean (SEM). Comparisons of samples to establish the statistical significance of difference were determined by the Mann-Whitney U-test or Wilcoxon signed-rank test, as indicated. All statistical analyses assumed a 2-sided significance level of 0.05. Data analyses were performed with Openstat software.

## Results

### Fibronectin as a substrate promotes the appearance of ECFC colonies earlier than collagen

**Figure 1:**
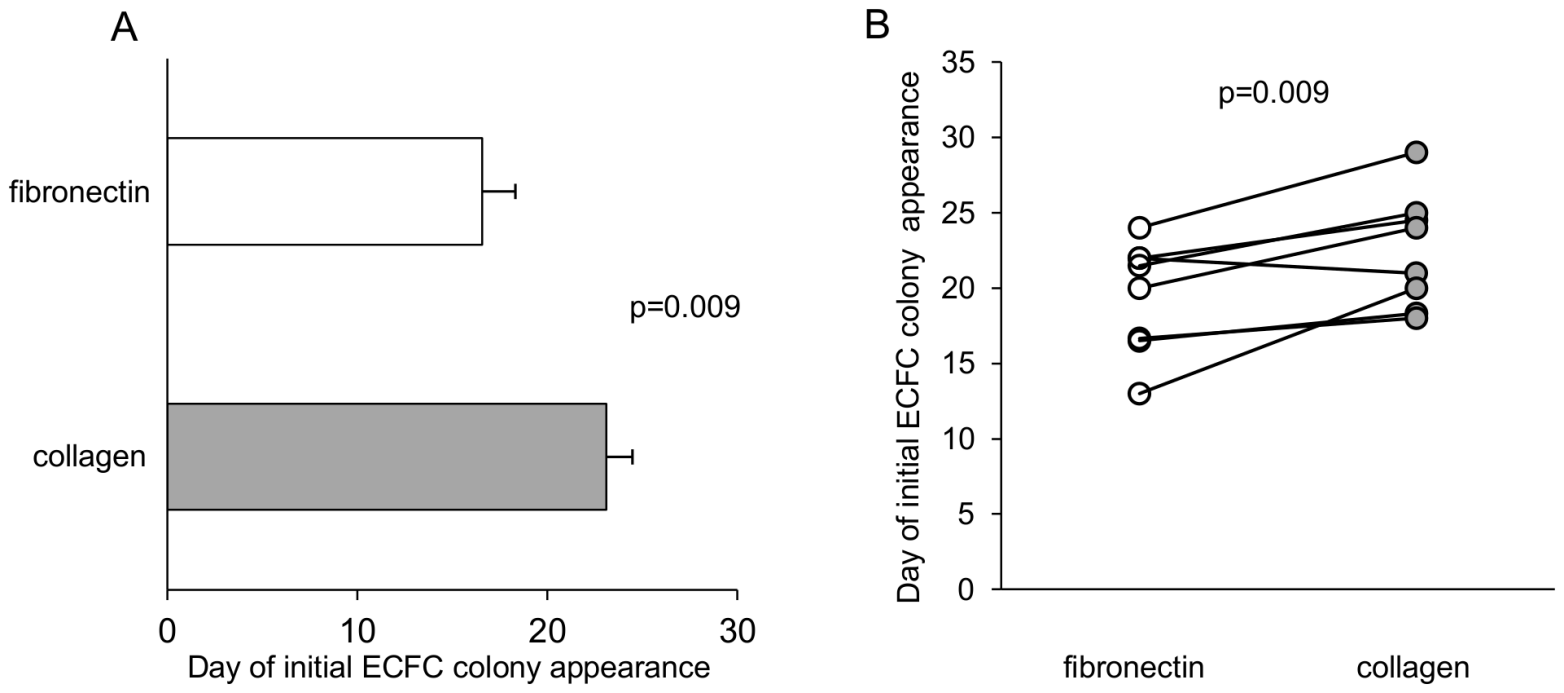
Effects of fibronectin and collagen on isolation of ECFC colonies. Fibronectin promoted the appearance of ECFC colonies earlier than collagen. (A) PBMCs from 9 donors were seeded on fibronectin-coated plates and PBMCs from other 9 donors were seeded on collagen-coated plates; ECFC colonies from PBMCs seeded on fibronectin (white bar) appeared earlier than those seeded on collagen (gray bar). p value calculated by the Mann-Whitney U-test. (B) PBMCs from 8 donors were divided into portions, with half of them seeded on fibronectin-coated plates and the rest seeded on collagen-coated plates; also in these paired experiments ECFC colonies from PBMCs seeded on fibronectin (white circles) appeared earlier than those seeded on collagen (gray circles). p value calculated by the Wilcoxon signed-rank test.

To assess whether the use of fibronectin rather than collagen as a substrate may differently affect the efficiency of isolation of ECFC colonies from peripheral blood, we compared the appearance of ECFC colonies obtained after PBMC seeding on either substrate. To this aim, we isolated PBMCs from 50 ml of peripheral venous blood from 18 healthy donors (9 men, 9 women; mean age 34±3 years). PBMCs from 9 donors were then seeded on fibronectin-coated plates and PBMCs from the other 9 donors were seeded on collagen-coated plates. A similar number of PBMCs was seeded in each condition (fibronectin: 69.2±9.5×10^6^ PBMCs vs collagen: 66.8±6.6, p = ns). As expected, colonies with cobblestone-like morphology appeared on both extracellular matrix protein substrates. At least one colony was obtained in 7 of 9 donors in both conditions, and similar frequencies of ECFC colonies were obtained on the two substrates (fibronectin: 3.5±1.1 colonies/10^8^ seeded PBMCs vs collagen: 3.3±0.7, p = ns). However, ECFC colonies from donors whose PBMCs were seeded on fibronectin appeared some days earlier than those seeded on collagen (16.6±1.7 days vs 23.1±1.4, p = 0.009) (Figure 1A).

To subtract possible bias due to interindividual variability, we repeated the same experiments comparing the influence of fibronectin and collagen on the efficiency of ECFC colony isolation starting from PBMCs obtained from a same donor. To this aim, we isolated PBMCs from 100 ml of peripheral venous blood from 8 healthy donors (4 men, 4 women; mean age 31±3 years). PBMCs from the same donor were divided into portions, with half of them seeded on fibronectin-coated plates and the rest seeded on collagen-coated plates. A mean of 75.0±9.2×10^6^ PBMCs were seeded in each condition. Also in these paired experiments, no difference was observed in the quantity of ECFC colonies obtained with the two substrates. In fact, at least one colony was obtained from all donors in both conditions, and the frequency of ECFC colonies did not differ between substrates (fibronectin: 3.0±0.6 colonies/10^8^ seeded PBMCs vs collagen: 3.5±0.9, p = ns). Similarly to what we observed in the unpaired experiments, also in these paired experiments ECFC colonies appeared few days earlier when PBMCs were seeded on fibronectin rather than collagen (19.4±1.3 days vs 22.5±1.3, p = 0.009) (Figure 1B). Once established, ECFC colonies obtained on both fibronectin and collagen increased rapidly in size, indicating a high proliferation rate. No differences were observed in colony size depending on the culture substrate.

### Collagen as a substrate sustains ECFC cell expansion more efficiently than fibronectin

**Figure 2:**
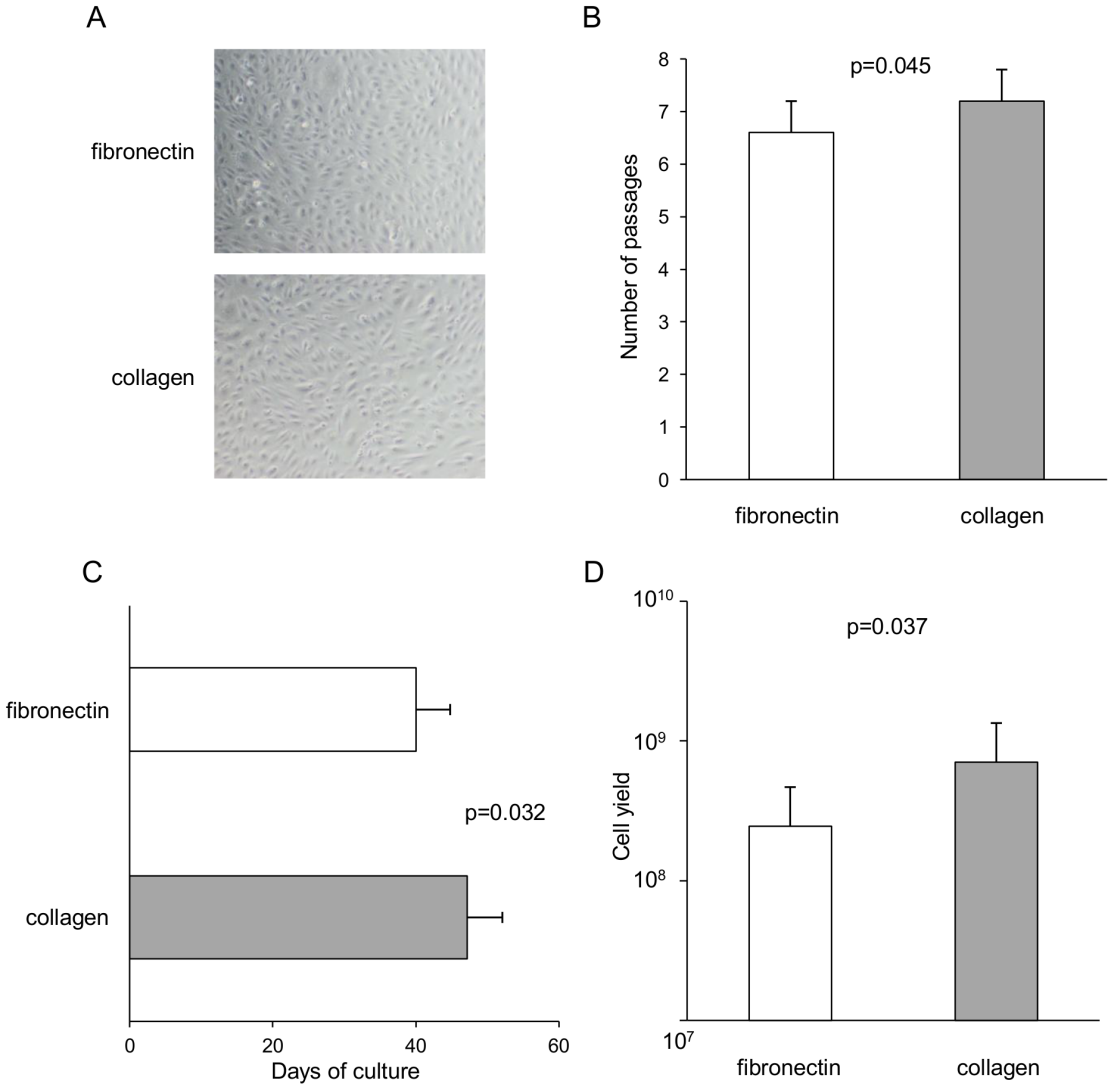
Effects of fibronectin and collagen on ECFC cell expansion. Collagen sustained cell expansion of ECFCs more efficiently than fibronectin. 14 ECFC colonies were divided into portions, with half of them seeded on fibronectin-coated plates and the rest seeded on collagen-coated plates. (A) Representative phase-contrast photographs showing that ECFCs expanded on fibronectin and collagen formed similar cobblestone-like monolayers with endothelial-like morphology. Photographs were obtained using an Olympus inverted microscope IX51, x10 magnification. ECFCs cultured on collagen (gray bar) compared with fibronectin (white bar) achieved (B) higher number of passages, (C) longer lifespan and (D) higher cell yields. p values calculated by the Wilcoxon signed-rank test.

We further asked whether the use of fibronectin rather than collagen as a culture substrate may differently affect the efficiency of cell expansion of ECFCs. To address this question, we compared the behaviour of ECFCs cultured on either substrate. To overcome the variability of cell growth rate commonly observed among different ECFC colonies, we selected 14 colonies isolated on fibronectin or collagen and, after their initial expansion to form an endothelial monolayer, we divided them into portions, with half of them seeded on fibronectin-coated plates and the rest seeded on collagen-coated plates. Independently from the substrate used for colony isolation, ECFCs cultured on both substrates underwent cell proliferation and could be serially passaged. During the passages the cells retained their endothelial-like morphology, forming cobblestone-like monolayers that were similar in the two culture conditions (Figure 2A). On both substrates ECFCs showed a finite replicative lifespan and could be passaged in some case up to passage 11 over a period of up to 70 days. However, ECFCs cultured on collagen achieved a significantly higher number of passages (7.2±0.6 vs 6.6±0.6, p = 0.045) (Figure 2B) and showed a longer lifespan before undergoing cell senescence (47.2±4.9 days vs 40.0±4.8, p = 0.032) (Figure 2C), compared with ECFCs cultured on fibronectin. More importantly, higher cell yields were obtained at the end of the culture when ECFCs were cultured on collagen rather than fibronectin (7.1×10^8^ cells vs 2.5×10^8^, p = 0.037) (Figure 2D).

### Cytokines produced by ECFCs may contribute to the increased cell expansion of ECFCs cultured on collagen

**Figure 3:**
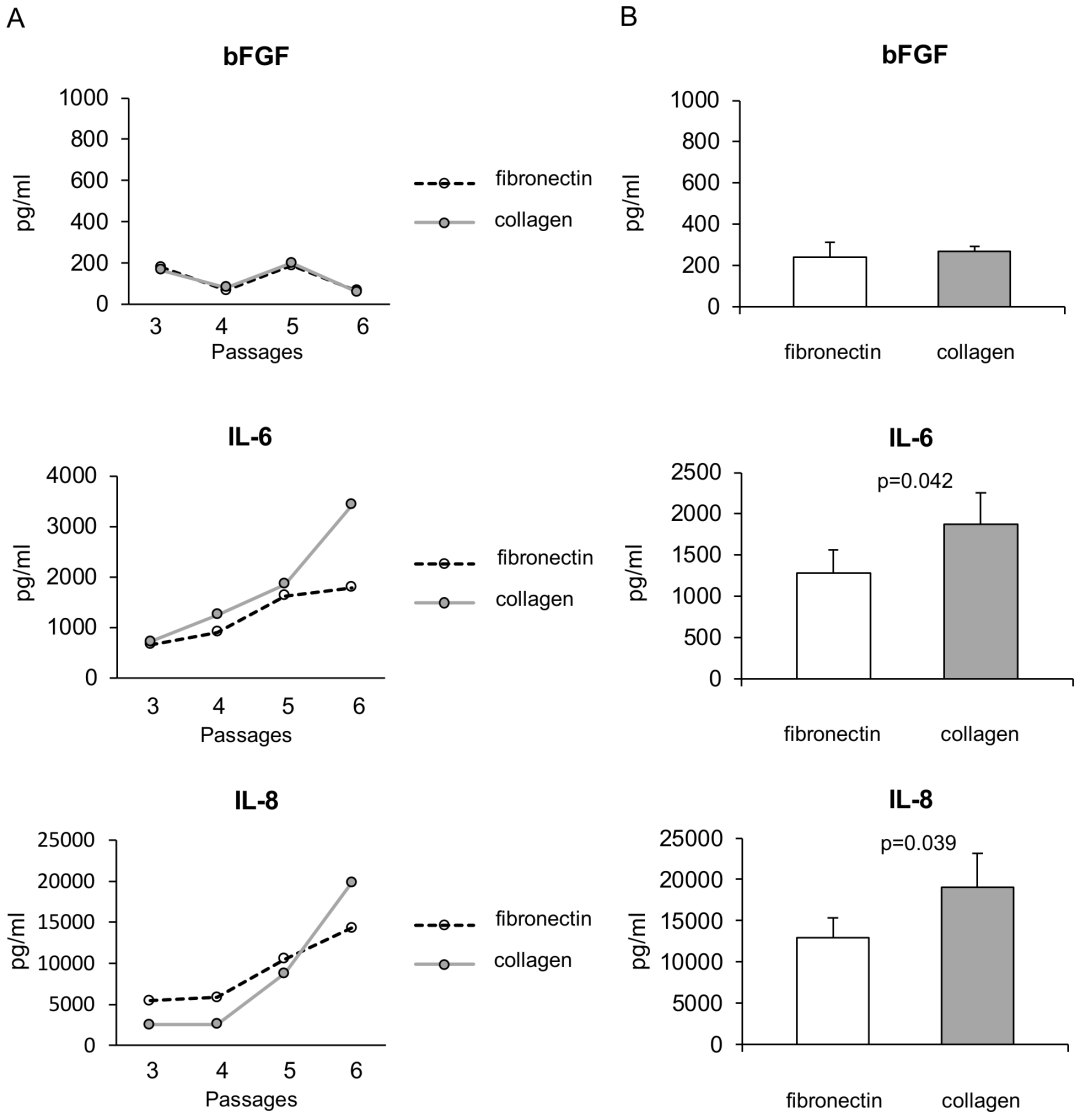
Effects of fibronectin and collagen on cytokine production by ECFCs. ECFCs expanded on collagen produced higher levels of IL-6 and IL-8 that ECFCs expanded on fibronectin. (A) Representative curves showing that the concentration of IL-6 and IL-8 in the supernatants of ECFCs expanded on fibronectin (white circles) and collagen (gray circles) tended to increase with increasing passages. (B) ECFCs expanded on collagen (gray bars) reached higher levels of IL-6 and IL-8, but not bFGF, than ECFCs cultured on fibronectin (white bars). The mean ± SEM of the highest cytokine concentration reached during culture of the same 14 ECFC colonies reported in Figure 2 is shown. p values calculated by the Wilcoxon signed-rank test.

To investigate the mechanisms possibly involved in sustaining the higher cell yield observed when ECFCs were cultured on collagen rather than fibronectin, we analysed in our cultures the main proangiogenic cytokines secreted by endothelial progenitor cells [37]. To this aim, we measured the levels of VEGF, bFGF, IL-6 and IL-8 in the supernatants recovered from each ECFC culture just before cell passaging. VEGF was undetectable at any passage in ECFC cultures expanded on any substrate, in accordance with a previous study [38]. Representative curves of the other cytokines measured during ECFC cultures are reported in Figure 3A, showing that the concentration of IL-6 and IL-8, but not bFGF, tended to increase with increasing passages and to be higher in ECFC cultures expanded on collagen. The comparison of the maximum levels of cytokines reached in the supernatants during ECFC culture further indicated that the levels of IL-6 and IL-8 were higher in ECFC cultures expanded on collagen rather than fibronectin (collagen vs fibronectin, IL-6: 1870±384 pg/ml vs 1287±276, p = 0.042; IL-8: 19029±4153 pg/ml vs 12916±2458, p = 0.039), while the levels of bFGF were similar in the two conditions (269±24 pg/ml vs 243±69, p = n.s.) (Figure 3B).

**Figure 4:**
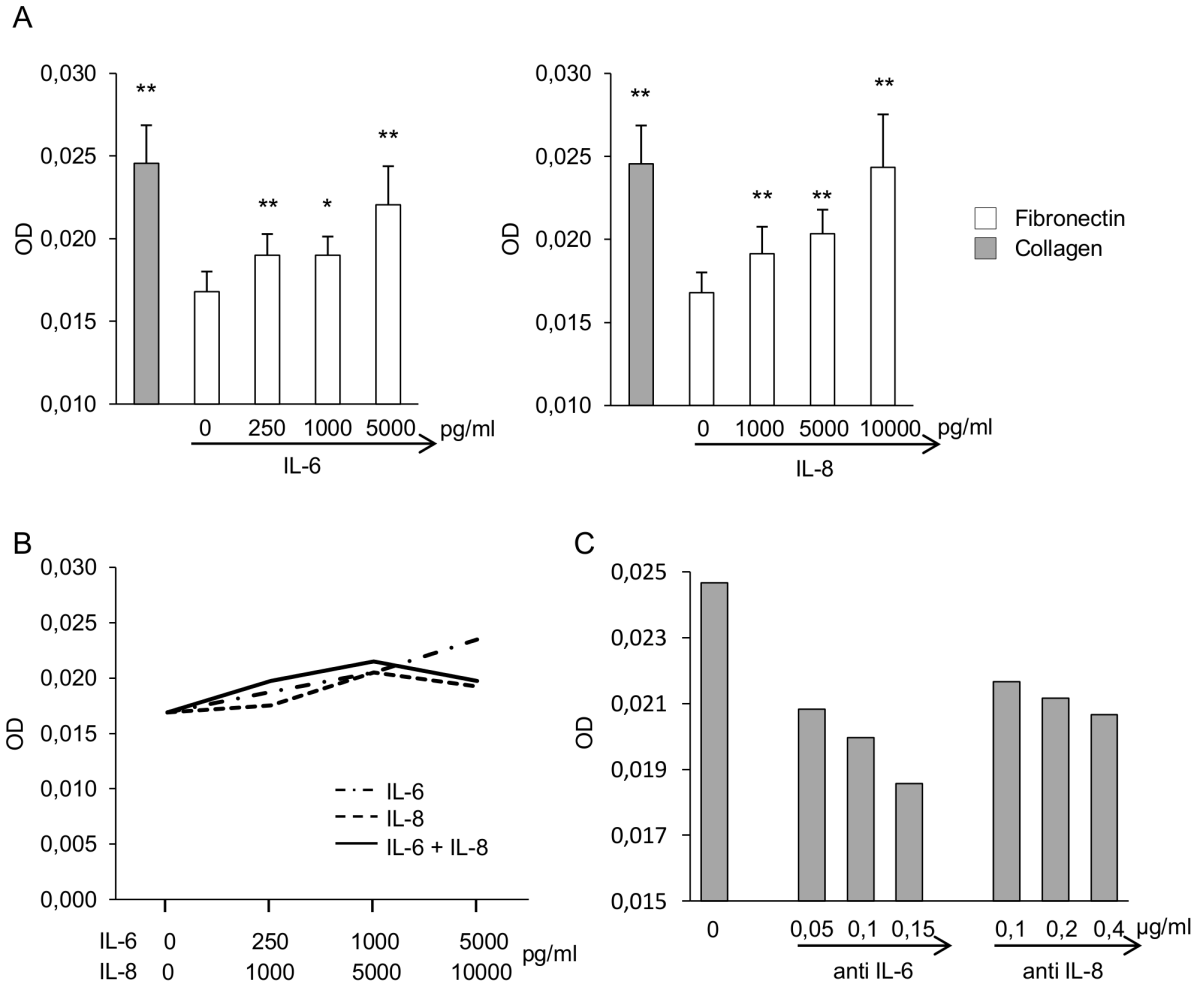
Effects of IL-6 and IL-8 on ECFC proliferation. IL-6 and IL-8 may account, at least in part, for the higher cell proliferation of ECFCs cultured on collagen as compared to fibronectin. Cell proliferation was assessed by the colorimetric CV assay, and expressed as OD. (A) Addition of IL-6 or IL-8 to ECFCs cultured on fibronectin increased cell proliferation in a dose-dependent manner. The mean ± SEM of 7 ECFC cultures is shown. *p = 0.046 and **p = 0.009 compared with untreated ECFCs cultured on fibronectin, as calculated by the Wilcoxon signed-rank test. (B) Addition of IL-6 and IL-8 together in the same culture did not further increase cell proliferation. One representative of three experiments is shown. (C) Addition of neutralizing anti-IL-6 or anti-IL-8 mAb to ECFCs cultured on collagen induced a dose-dependent reduction of cell proliferation. One representative of three experiments is shown.

To further assess whether IL-6 and IL-8 were responsible indeed for the difference in ECFC expansion observed on different substrates, we investigated whether addition of these cytokines to ECFCs cultured on fibronectin could recapitulate the behaviour of ECFCs cultured on collagen. To this aim we analysed the effects of IL-6 and IL-8, added at concentrations similar to those measured in culture supernatants, on ECFC proliferation. Cell proliferation was assessed by the colorimetric CV assay, and expressed as optical density (OD). As shown in Figure 4A, cell proliferation of ECFCs cultured on collagen was significantly higher than proliferation of ECFCs cultured on fibronectin, as expected. Both IL-6 and IL-8 added to ECFCs cultured on fibronectin induced a significant and dose-dependent increase in cell proliferation, inducing OD values similar to those of ECFCs cultured on collagen. The addition of IL-6 and IL-8 together in the same culture did not further increase cell proliferation (Figure 4B). To further define the role of IL-6 and IL-8 in sustaining the higher expansion of ECFCs on collagen, we performed inhibition experiments with blocking antibodies. As shown in Figure 4C, both anti-IL-6 and anti-IL-8 mAbs added to ECFCs cultured on collagen induced a dose-dependent reduction of cell proliferation.

### ECFCs expanded on fibronectin or collagen have similar immunophenotype and ability for in vitro tubulogenesis

**Figure 5:**
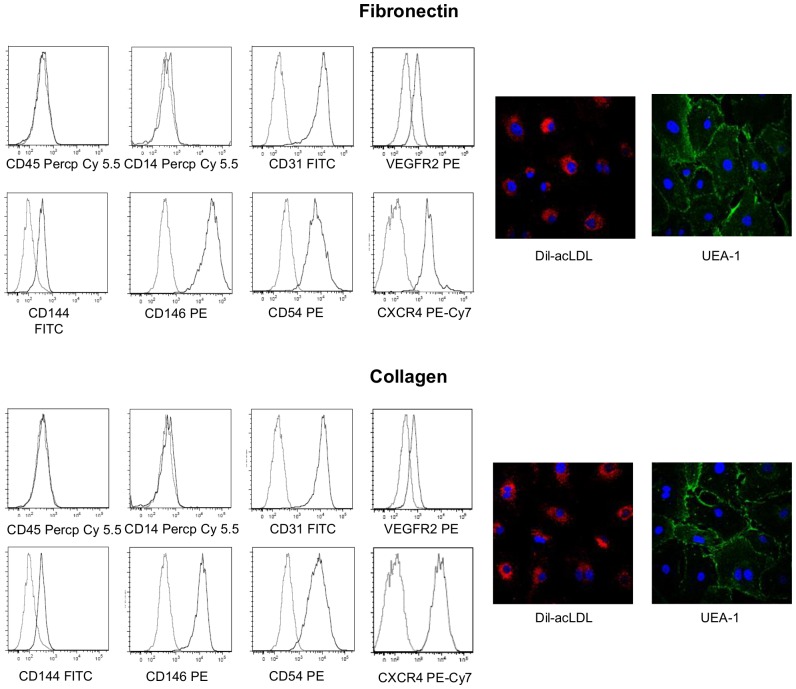
Effects of fibronectin and collagen on the immunophenotype of ECFCs. ECFCs expanded on fibronectin or collagen showed a similar immunophenotype. On the left are reported flow cytometric histograms showing that, independently from the substrate used for cell culture, ECFCs did not express CD45 and CD14, while they expressed at similar levels CD31, VEGFR2, CD144, CD146, CD54 and CXCR4. On the right are reported immunofluorescence photographs showing that ECFCs expanded on both substrates incorporated Dil-ac-LDL and bound lectin UEA-1. Photographs were obtained using an an Olympus Fluoview FV1000 confocal microscope, x40 magnification. One of three representative experiments is reported.

We then asked whether coating of culture plates with fibronectin rather collagen as an extracellular matrix protein may differently affect the immunophenotype or functional properties of the expanded cells. To this aim, we compared early passaged (3–5) ECFCs expanded on fibronectin with equally passaged ECFCs expanded on collagen. As shown in Figure 5, ECFCs cultured on both substrates had a comparable endothelial phenotype. In particular, immunophenotyping by flow cytometry revealed that in both cases ECFCs lacked the hematopoietic markers CD45 and CD14 and expressed the endothelial cell surface antigens CD31, VEGFR2, CD144, CD146, CD54 and CXCR4 to a similar extent. Furthermore, ECFCs expanded on both substrates incorporated Dil-ac-LDL and bound lectin UEA-1.

**Figure 6:**
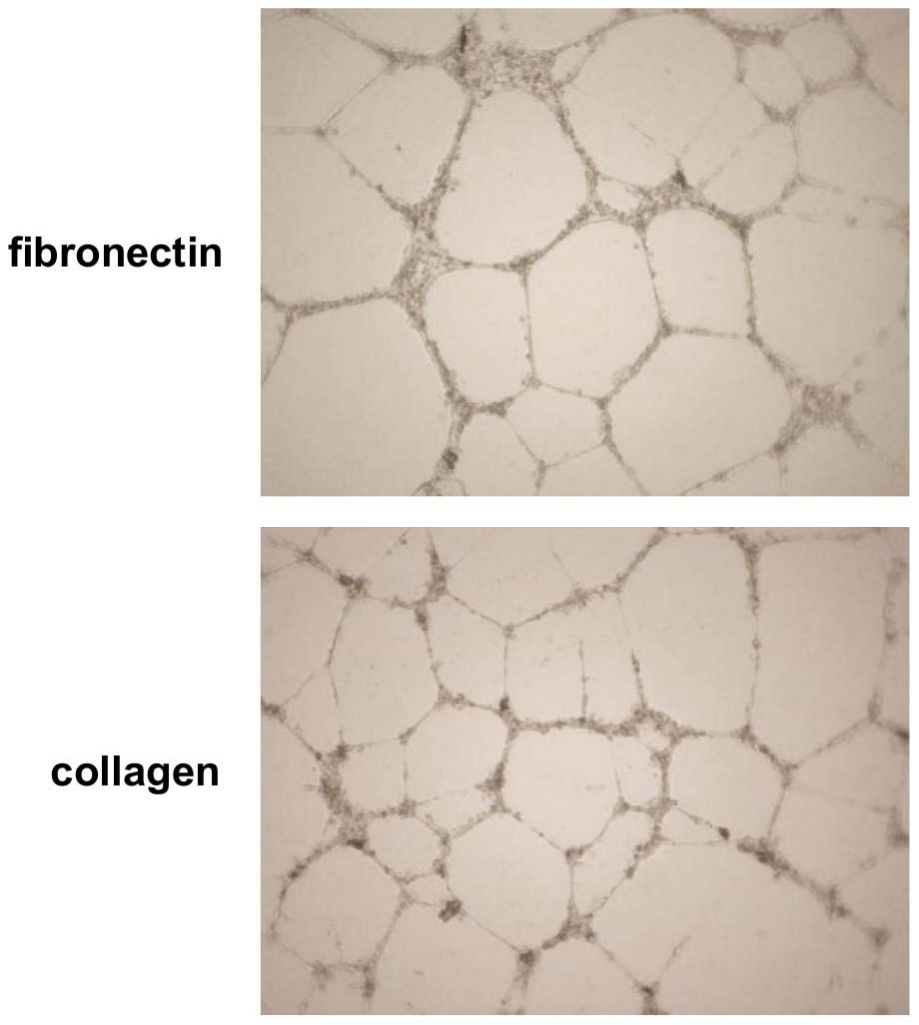
Effects of fibronectin and collagen on the ability of ECFCs for in vitro tubulogenesis. ECFCs expanded on fibronectin or collagen showed a similar ability to form capillary-like structures in vitro. Independently from the substrate used for cell culture, ECFCs cultured in Matrigel gave rise within 12 hours of incubation to vascular structures. Representative phase-contrast photographs showing that the capillary-like structures formed by ECFCs cultured on fibronectin and collagen were similar. Photographs were obtained using an Olympus inverted microscope IX51, x4 magnification.

As shown in Figure 6, the Matrigel capillary tube formation assay demonstrated that ECFCs expanded on both substrates were similarly able to form capillary-like structures in vitro.

## Discussion

In this study, we provide evidence that fibronectin and collagen used for culture surface coating differently affect the efficiency of ECFC isolation and expansion. This is an important issue. In fact, although ECFCs are increasingly being studied in various diseases because of their potential for clinical translation, yet experimental procedures for their ex vivo culture still lack standardization. In particular, either fibronectin or collagen are commonly used as coating substrates to obtain cells that have all the features needed to be defined ECFCs [21]–[32], [39], without any evidence for advantages of one substrate over the other. Our results clearly confirmed that both substrates allow the isolation and expansion of ECFCs with frequency of obtained colonies, cell growth rates, cell yields and cell features similar to those reported by other Authors [27], [29], [40]–[43]. However, by analysing the impact of fibronectin compared with collagen as the only variable of the experimental protocol, we could demonstrate that there were some differences between substrates. Moreover, by analysing separately the phase of isolation of ECFC colonies appearing from seeded PBMCs, and the following phase of cell propagation of ECFCs, we could demonstrate some advantages of the two substrates within individual steps of the entire process.

The results obtained in the first step of the process indicated that fibronectin sustained the isolation of ECFC colonies more efficiently than collagen. In fact, maintaining constant all the other experimental variables, PBMCs seeded on fibronectin gave rise to ECFC colonies almost one week earlier than PBMCs seeded on collagen. Notably, this advantage of fibronectin-coating was confirmed in the experiments where the comparison between substrates was performed within the same subject, thus overcoming differences possibly due to interindividual variability. We suggest that the shorter time of initial cluster appearance supported by fibronectin may be advantageous, as it may help reduce time-and reagent-consuming procedures in the lab and may allow getting results faster.

On the other hand, the results obtained in the second step of the process indicated that collagen sustained more efficiently than fibronectin the cell propagation of ECFCs. In fact, the comparison of parallel cell cultures, started from the same colony and performed in identical experimental conditions apart from the substrate used for plastic coating, demonstrated that higher cell yields were obtained when ECFCs were cultured on collagen, likely related to the longer survival of cell cultures and the higher cell proliferation observed on this substrate. Notably, the supernatants obtained from ECFCs cultured on collagen contained higher levels of IL-6 and IL-8, angiogenic cytokines that have been demonstrated to directly promote the survival of endothelial cells, as well [39], [44]. It may be possible that the higher levels of IL-6 and IL-8 may derive from the higher cell yields and longer lifespan of ECFCs cultured on collagen. However, the results obtained in the experiments of cytokine supplementation and blocking seem to support that IL-6 and IL-8 may account, at least in part, for the differences in the expansion of ECFCs on the different substrates. Apart from these differences in cytokine production, our results clearly indicated that ECFCs expanded on fibronectin and collagen shared similar ability for in vitro tubulogenesis and similar phenotype. It is important to note that also CXCR4, receptor for stromal-derived cell factor-1 with a key role in selective migration and recruitment of endothelial progenitor cells into the sites of blood vessel formation and repair [14], [45], was similarly expressed on ECFCs cultured on the two substrates. This is a crucial issue, because ECFCs are increasingly used in studies of in vivo vasculogenesis, where the ability of these cells to adequately localize into the sites of tissue injury is indeed a crucial prerequisite. Notably, controversial results on the expression of CXCR4 by ECFCs have been reported, possibly related to the source of PBMCs used for ECFC culture [26], [46], [47]. All together, the results obtained analysing the expansion phase of ECFC culture demonstrated that fibronectin and collagen allow the propagation of ECFCs with very similar features. However, the use of collagen may be advantageous as it permits to obtain a higher number of cells to be used for subsequent experiments. An additional advantage deriving from the use of collagen for plastic coating in the expansion phase may be economic, as collagen is about twenty times cheaper than fibronectin, and the consumption of coating substrate during ECFC expansion may be huge.

We conclude from this study that, although both fibronectin and collagen are appropriate to sustain the entire process of ECFC culture, colony isolation on fibronectin followed by cell expansion on collagen may represent the most efficient strategy to obtain ECFCs from adult peripheral blood samples.
